# Electrochemotherapy and Simultaneous Photodynamic Bone Stabilization of Upper Limbs in Metastatic Renal Cancer Disease: Case Report and Literature Review

**DOI:** 10.1155/2020/8408943

**Published:** 2020-10-14

**Authors:** Ugo Albertini, Andrea Conti, Nicola Ratto, Pietro Pellegrino, Michele Boffano, Raimondo Piana

**Affiliations:** ^1^Oncologic Orthopaedic Division, Department of Orthopaedic and Traumatology, Orthopaedic and Trauma Center, Città Della Salute e Della Scienza,Via Gianfranco Zuretti 29, 10126 Turin, Italy; ^2^University of Turin, Italy-Via Gianfranco Zuretti 29, Turin 10126, Italy

## Abstract

**Introduction:**

Metastatic bone disease represents a systemic pathology that heavily affects the quality of life of oncologic patients causing pain and functional disability. *Methodology*. We present the case of a patient with a history of renal cell cancer presenting pathologic fractures of both humeri and proximal right radius.

**Results:**

After a careful multidisciplinary approach, an adjuvant anticancer therapy and a photodynamic bone stabilization procedure were performed with a minimally invasive technique aiming to minimize pain and local disease progression, while restoring functional autonomy and improving the patient's quality of life. Electrochemotherapy was delivered on the lytic bone lesions with extraskeletal involvement of the proximal left humerus and the proximal right radius, and then polymeric bone stabilization was performed on both humeri. At two months of follow-up, the patient presented satisfactory functional scores (MSTS score: 12/30 bilaterally; DASH scores: 46.7/100 for the right side and 48.3/100 for the left one), and pain was well controlled with opioid analgesics. Radiographs showed good results in terms of ossification of lytic bone lesions and durability of polymeric stabilization. At four months of follow-up, the patient reported a stable clinical scenario. Six months after surgery, due to extremely poor prognosis after the progression of primary disease, the patient was referred to palliative care and died shortly thereafter.

**Conclusion:**

Over the last decade, the management of metastatic bone disease has changed. Low-toxicity and minimally invasive procedures such as electrochemotherapy and polymeric bone stabilization might be performed concomitantly in selected patients, as an alternative to radiation therapy and to more demanding surgical procedures such as plating and adjuvant cementing.

## 1. Introduction

The most common cause of destructive bone lesions is metastatic cancer. Bone is the third most common site for metastasis after lung and liver, while prostate, breast, lung, renal, and thyroid cancer account for around 80% of all skeletal metastases [1]. Bone metastases are commonly associated with a high morbidity burden that heavily affects the quality of life of oncologic patients. Severe pain can be experienced due to bone pain and compression of spinal cord or nerve roots, and it requires adequate multidisciplinary treatment that can include narcotic analgesics, chemotherapy, radiation therapy, and bisphosphonate. Surgery is mainly indicated in nonresponding tumors or when pain is not controlled with medical treatment. Around 10–25% of patients present with an impending or actual pathologic fracture [2], often requiring surgery and representing a catastrophic event for these patients. In addition to the fracture-related complications that could lead to immobilization, possible surgery, and consequent suspension of tumor-related treatments, other possible negative effects such as contamination of adjacent joint, soft tissue, nerves, and vessels by hematoma formation, or distant hematogenous dissemination due to microcirculation damages must be considered. Around 30% of the patients with renal cell cancer eventually develop bone metastases, and these are associated with worse overall survival [3]. Renal cancer bone metastases are usually lytic and highly hypervascular and cause technical issues especially during surgical management when the surgeon must face difficulties in achieving bone stability for pain control. Preoperative embolization is often required to minimize blood loss [4–6], and whenever radical surgery is not indicated, minimally invasive palliative treatments, such as electrochemotherapy, radiofrequency ablation, high intensity focus ultrasound, and photodynamic polymer bone stabilization, have become more and more popular [7–10].

At our institution, an Orthopedic Oncologic Department of a national reference center for musculoskeletal tumor surgery, the concomitant use of electrochemotherapy and a polymeric intramedullary stabilization system was chosen to treat multiple bone metastases of upper limbs in a multimetastatic renal cancer case.

## 2. Case Presentation

**Figure 1:**
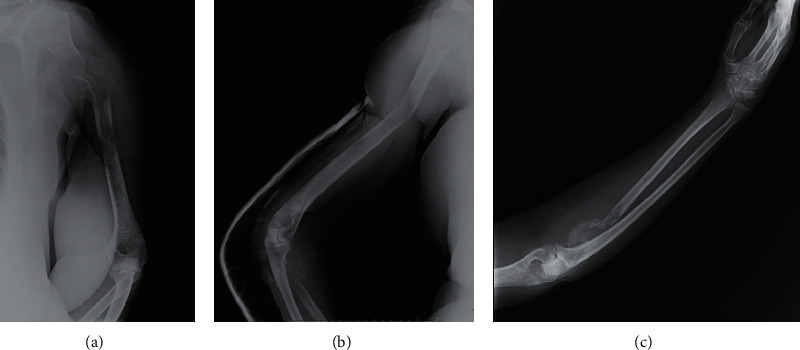
Preoperative radiographs demonstrating simultaneous lytic lesions and pathologic fractures of upper limbs secondary to a renal cell cancer.

**Figure 2:**
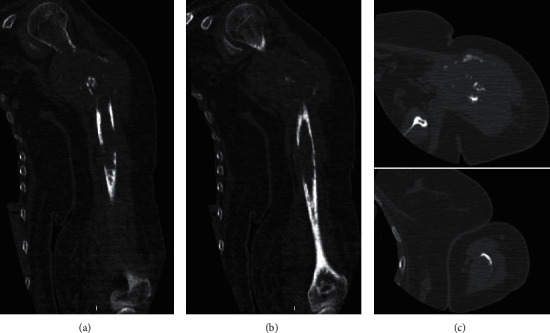
Preoperative CT scans demonstrating a large extraskeletal mass in the proximal left humerus causing a pathologic fracture, while a second lytic lesion is visible distally. On the right side: in the upper and lower corner, axial CT scans of the proximal and distal lesions are visible, respectively. (CT: computed tomography).

**Figure 3:**
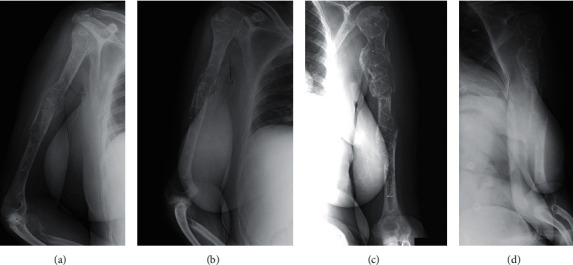
(a, b) Two radiographs showing bone healing of the pathologic fracture of the right humerus at two months of follow-up. (c, d) Two radiographs showing bone healing of the pathologic fracture of the left humerus at two months of follow-up.

**Figure 4:**
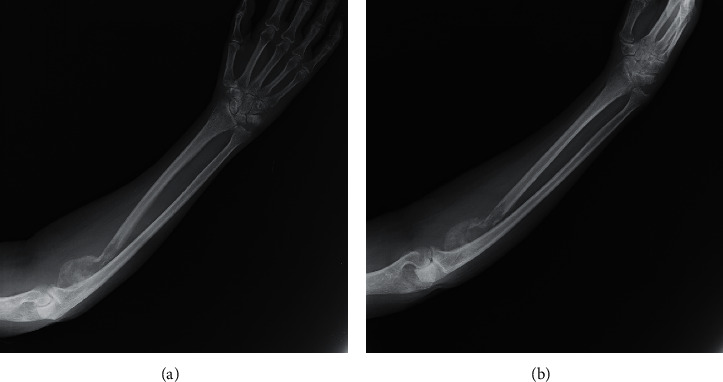
Two radiographs showing bone healing of the pathologic fracture of the right radius at two months of follow-up.

A nonsmoker 60-year-old woman with a history of hiatal hernia with Barrett's esophagus and depression was admitted to the Emergency Department due to persistent fever during the last month. Blood tests, ultrasounds (US), and computed tomography (CT) imaging showed an 8 cm right mesorenal cancer with infiltration of the perirenal fat, urinary collecting system, and renal sinus. Moreover, the presence of floating thrombus in the right renal vein up to the inferior caval vein was detected, while the absence of a cleavage plane with the lower face of the liver was reported. Radical right nephrectomy and homolateral renal vein thrombectomy were performed after 15 days from diagnosis. Histological examination revealed a pT3a G4 N1 clear cell renal cancer with extensive pleomorphic and rhabdoid aspects. A week after surgery, the patient suffered from left pulmonary thromboembolism, which required anticoagulant therapy. An acute pain at the left arm and right forearm occurred two months after surgery with no history of relevant trauma. Pathologic fractures were found in both the diaphysis of the left humerus and the proximal metaphysis of the right radius. The patient showed pain (NRS score 8/10 [11]), swelling, and both upper limbs' complete limitation of movement plus systemic fatigue and poor appetite. No fever was detected. The peripheral blood erythrocyte count was below 3 × 10^12^/L, and hemoglobin was around 95 g/L. The Karnofsky score was 30. After hospital admission, the patient received parenteral nutrition, analgesics, and other symptomatic treatments. Oral anticoagulant therapy was suspended and replaced with subcutaneous low-molecular-weight heparin (LMWH) therapy. After a multidisciplinary team discussion, the patient was addressed for surgery. While undergoing the routine preoperative workups, a spontaneous diaphyseal fracture of the right humerus occurred. Preoperative radiographs (as in Figure 1) demonstrated the fractures and wide lytic lesions involving the left humerus and the proximal right radius and confirmed the pathologic fracture of the right humerus. A CT scan of the thorax and abdomen was performed, and no secondary lesions were detected apart from the bone localizations. An additional CT scan of the upper limbs was performed to refine the surgical indication (Figure 2). Large osteolytic lesions with soft tissue invasion, involving the proximal left humerus and the proximal right radius were noticed. Our approach consisted of delivering a local adjuvant therapy on the lesions of the left humerus and right radius with electrochemotherapy using Cliniporator VITAE® technology (IGEA Spa; Carpi, MO, Italy). Thus, closed reduction and intramedullary fixation using a photodynamic polymeric stabilization system (IlluminOss® -IlluminOss Medical GmbH; Hilden, Germany) of both humeri was performed. The patient underwent general anesthesia and the entire procedure in a supine position. To treat both intraosseous and extraskeletal components of the lesions, electrochemotherapy was applied as follows: 4 and 7 single long (16 cm) needle VGD-1830T16 electrodes (variable geometry) with 1.8 mm in diameter and 3 cm active part were positioned around the right radial lesion and the left humeral lesion, respectively, and then the patient received a bolus of bleomycin (Bleomycin Nippon-Kayaku, Sanofi-Aventis, vials 15 mg, 15,000 UI/m^2^ of body surface area). Eight minutes after the infusion, to allow the drug diffusion into the tumour tissues, electric pulses were applied (electroporation) according to the standard operating procedures. A single train of eight electric pulses of 100 *µ*s of duration at 1000 V/cm was generated by the Cliniporator Vitae® (IGEA spa Carpi). Afterwards, a small incision was performed anterolaterally to the acromion process of the left scapula, the deltoid muscle was split longitudinally to expose the subdeltoid bursa, and then the supraspinatus tendon was incised in line with its fibers. The fracture was reduced with the aid of an intramedullary guidewire under fluoroscopic guidance and the canal prepared with flexible cannulated reamers. Thus, the Dacron® balloon catheter was inserted, and the monomer was infused. The light source was finally used to activate the monomer, the tissues were closed in layers, and the correct position of the implant was checked by fluoroscopy. When the stability of the left humerus was reached, the contralateral fracture was internally stabilized with the same photodynamic bone stabilization system. After the operation, the right arm was immobilized with a long splint for 20 days, while arm slings were positioned bilaterally. Passive mobilization was prescribed from the first day following the operation, active elevation of the arms was restricted for the first 20 days, while lifting and strength-training were prohibited for a period of six weeks. The postoperative period was complicated by anemia, and five blood units were given to the patient. The pain was controlled with the administration of oral oxycodone/naloxone 5 mg/2.5 mg combination every 12 hours. After oncological examination, 24 days after surgery, a systemic treatment with pazopanib 400 mg once a day was prescribed, and the patient was discharged to an outpatient rehabilitative long-term care ward. Two months following the operation, a clinical follow-up reported that all surgical scars were well healed, and the range of motion was acceptable for low-impact daily activities. Anterior elevation and abduction of both shoulders were around 90°, internal rotation was limited to the buttock, while the elbows demonstrated full range of motion bilaterally. While slight pain (NRS score 3/10) was reported during the mobilization of the left shoulder, no pain was evocable by palpation of the treated bone lesions or at rest (NRS score 0/10). Nonetheless, the patient continued the same opioid dosage as at hospital discharge. MSTS score [12] was 12/30 (40%) bilaterally, while DASH scores [13] were 46.7 and 48.3 for the right and left side, respectively. Along with the clinical examination, radiographs were performed (Figures 3 and 4) and showed no secondary mobilization of the implants, an initial callus formation, and new ossification processes. A radiographic partial response with a reduction of approximately 30% of the lesions' diameters was reported (RECIST criteria [14]). In conjunction with the oncologic team, the administration of pazopanib was confirmed, and a further clinical and radiological follow-up was scheduled at 6 months from surgery. At 4 months after surgery, the patient was contacted by telephone and reported substantial stability of the clinical scenario with well-controlled pain by mild opioid analgesics. At 6 months after surgery, a follow-up chest and abdomen CT exam detected lymph node infiltration of the caudate lobe of the liver and bone progression of disease. Furthermore, neck and supraclavicular lymph node metastases were reported. The oncologist suspended the treatment with pazopanib, and palliative care was initiated. The patient died within a month. This case report was approved by the United Ethical Committee of “AOU Città della Salute *e* della Scienza,” Turin, Italy, in accordance with the Declaration of Helsinki. The patient signed an informed consent form and authorized the use of data for research purposes.

## 3. Discussion

The treatment of the multimetastatic patients has changed radically over the last 20 years: several surgical treatments have been introduced to control pain, maintain independent living, and improve the quality of life, along with improved medical treatments that target the prevention of tumor progression. Surgery is typically employed for impending fracture or for actual pathologic fractures, but when surgery is not indicated, fractionated radiation therapy (8 Gy/1f or 30 Gy/10f) is offered as first-line treatment. However, radiation therapy is not effective in around 30% of cases [15], and reaching of the maximum dose limits its usage. Moreover, patients might suffer from intolerance in the surrounding tissues and from weakening of the healthy bone [7].To the best of the authors' knowledge, this is the first case report of a patient treated with internal fixation of pathologic fractures with an intramedullary polymeric stabilization system combined with simultaneous electrochemotherapy of upper limbs. The rationale was to apply adjuvant therapy to both the intraosseous and extraskeletal components to control tumor progression and pain, while providing immediate stability of the pathologic fractures with a minimally invasive surgical technique. In case of multimetastatic bone disease, the main objective of palliative treatments is to restore a partial/complete function controlling the pain and not always to reduce lesion size dimensions. Electrochemotherapy is based on the local intake of a cytotoxic chemotherapeutic agent (usually bleomycin) by means of electric pulses delivered to the tumor nodule via suitable sets of electrodes. Changes to the cell membrane potential determine the establishment of a transient passage: electroporation is induced, and water and charged molecules, such as some anticancer drugs, can pass through the cell membrane's newly formed pores, into the cells' interior. Such pores are established rapidly and disappear within minutes depending on the electric field amplitude [16]. In vivo studies [17–19] demonstrated that, after exposure to the electric field, the tumor's blood flow decreases up to 80% for about 24 hours allowing the cytotoxic agent to stay within the tumor for several hours and possibly determining a vascular-disrupting effect on the targeted cells. Electrochemotherapy's range of applications is extensive and includes cutaneous and subcutaneous metastases, melanoma, nonmelanoma skin cancer, soft-tissue sarcoma, and liver and bone metastases; in addition, some clinical trials have been conducted on selected primary tumors [20]. Even if the treatment of bone metastases and soft tissue masses with electroporation-based therapies is relatively recent [17, 21], good outcomes have been recorded in terms of pain control and tumor necrosis. A phase II clinical study [15] conducted on 29 patients affected by painful bone metastatic disease showed an improvement on pain control or a decrease in analgesic consumption in 84% of the cases, while a local progression was detected in around 7% of the patients. No patient suffered from intolerance to bleomycin; local complications were rare accounting for skin ulceration and necrosis in previously irradiated skin and a neurogenic bladder after the third treatment in a large lesion involving the sacrum. Overall, electrochemotherapy is safe and feasible in well-selected patients with multimetastatic bone disease and provides good results on pain control and local disease progression. It also allows treating tumor masses and nodules in the proximity of noble structures such as vessels and nerves as the treatment does not employ tissue heating [20]. Electrochemotherapy is currently in use at the referral centers for musculoskeletal surgery in Italy, while the Italian Society of Orthopedics and Traumatology (SIOT) has included it in the guidelines for the management of unresectable sacral tumors [22]. Also, a registry named ReinBONE (Registry on Electrochemotherapy in Bone), which is promoted by the Study Group for Bone Metastasis of the SIOT, is at present collecting clinical data on the treated cases. The photodynamic bone stabilization system is a recent, minimally invasive surgical technique that allows surgeons to repair bone fractures using alight-curable polymer contained within an inflatable balloon catheter [23]. This technique allows the time to obtain a proper reduction of the fracture before hardening the polymer, unlike polymethylmethacrylate (PMMA) cement: once correct alignment, rotational stability, and bone length have been restored, the visible light curing system is introduced to rapidly polymerize the liquid in the balloon to form a durable, hardened stabilizing rod. The ability of the system to get into contact with the cortical bone, filling the medullary space, significantly reduces the rotatory instability of the traditional intramedullary devices that require the use of further hardware, such as screws, to provide stability [24, 25]. Also, if necessary, the hardened polymer can be used as a substrate for supplemental osteosynthesis as screws can be inserted as in conventional nails, to provide supplemental stability [26]. A preliminary study reported no complications in the treatment of 36 osteoporotic and metastatic fractures of non-weight-bearing bones apart from one surgical revision of a humeral fracture [23]. To validate the initial studies, a European Union Registry has been set up: the first results reported achievement of procedural success, and no removal or revision of implants was required in 149 fractures treated with the polymeric rod [10]. Recently, a prospective study on 33 patients with traumatic humeral fractures treated with the polymeric rod showed a complete healing in the whole sample of patients, with good pain control and satisfying functional outcomes. The procedure's complication rate was around 35% which is not higher than what was reported in literature for other stabilization systems [26]. A comparison study among cemental plate fixation, intramedullary nailing, and photodynamic stabilization in the treatment of 105 malignant pathologic humeral fractures was conducted. No significant differences were registered in reoperation rates, but the rate of broken implants was significantly higher in patients treated with a polymeric rod [27]. Although the photodynamic polymer stabilization system is usually indicated for metastatic and osteoporotic fractures of non-weight-bearing bones fractures, several other uses are reported in the literature such as compassionate stabilization of femoral fractures in nonambulant patients [23] and surgical augmentation of acetabular and femoral fractures in patients with osteogenesis imperfecta [28, 29]. Also, a preliminary study on sheep provided encouraging results in the treatment of weight-bearing bone fractures [30]. The system is contraindicated in active or not completely healed infections, even if studies have been carried out on the antimicrobial effect of the light used for the polymerization of the system [31].

## 4. Conclusion

The management of a patient with a multimetastatic bone disease benefits a multidisciplinary approach. Whenever medical or radiation therapy is neither effective nor indicated, several palliative surgeries can be proposed to the patient. Minimally invasive and low-toxicity procedures such as bone electrochemotherapy and polymeric bone stabilization might be performed concomitantly in selected patients, in an attempt to synergize their benefits. Although clinical and functional outcomes of the above case report were satisfactory to improve the quality of life, further larger studies on combination of techniques could help surgeons make more conscious decisions.

## Data Availability

The radiographical imaging and data used to support the findings of this study are included within the article.
